# Route of Zika virus infection in *Aedes aegypti* by transmission electron microscopy

**DOI:** 10.1186/s12866-021-02366-0

**Published:** 2021-10-30

**Authors:** Thayane da Encarnação Sá-Guimarães, Tiago Souza Salles, Carlucio Rocha dos Santos, Monica Ferreira Moreira, Wanderley de Souza, Lucio Ayres Caldas

**Affiliations:** 1 Departamento de Bioquímica, Instituto de Química, Universidade Federal do Rio de Janeiro, CEP 21941-909 Rio de Janeiro, RJ Brazil; 2 Instituto Nacional de Ciência e Tecnologia em Entomologia Molecular, CEP 21941-902 Rio de Janeiro, RJ Brazil; 3 Laboratório de Fisiologia e Controle de Artrópodes Vetores, Instituto Oswaldo Cruz, CEP 21040-900 Fiocruz, Rio de Janeiro, RJ Brazil; 4 Universidade Federal do Rio de Janeiro, Centro Nacional de Biologia Estrutural e Bioimagem (CENABIO), CEP 21941-902 Rio de Janeiro, RJ Brazil; 5 Instituto de Biofísica Carlos Chagas Filho, Laboratório de Ultraestrutura Celular Hertha Meyer, Universidade Federal do Rio de Janeiro, CEP 21941-902 Rio de Janeiro, RJ Brazil; 6 Duque de Caxias, Universidade Federal do Rio de Janeiro, Núcleo Multidisciplinar de Pesquisa UFRJ-Xerém em Biologia – NUMPEX-BIO, RJ CEP: 25265-970 Rio de Janeiro, Brazil

**Keywords:** Zika virus, Aedes aegypti, Transmission Electron Microscopy, Eggs, Ovary, Vertical transmission

## Abstract

**Background:**

Zika fever has been a global health security threat, especially in the tropical and subtropical regions where most of the cases occur. The disease is caused by Zika virus (ZIKV), which belongs to the family *Flaviviridae*, genus Flavivirus. The virus is transmitted by *Aedes* mosquitoes, mostly by *Aedes aegypti*, during its blood meal. In this study we present a descriptive analysis, by transmission electron microscopy (TEM), of ZIKV infection in *A. aegypti* elected tissues at the 3rd day of infection. ZIKV vertical transmission experiments by oral infection were conducted to explore an offspring of natural infection.

**Results:**

Gut and ovary tissues harbored a higher number of viral particles. The ZIKV genome was also detected, by RT-qPCR technique, in the organism of orally infected female mosquitoes and in their eggs laid.

**Conclusions:**

The data obtained suggest that the ovary is an organ susceptible to be infected with ZIKV and that virus can be transmitted from mother to a fraction of the progeny.

## Background

Currently, zika fever has been a cause of worldwide health warning, especially in tropical and subtropical regions, where most of the cases have occurred [1, 2]. The disease is caused by Zika virus (ZIKV), which belongs to the *Flaviviridae* family, genus *Flavivirus* [3]. This virus was initially isolated in 1947 from the blood of sentinel monkeys for yellow fever in Uganda [4]. The first case of human infection was described in a 10-year-old child from Nigeria in 1954 [5]. In 2007, the first major outbreak of zika fever occurred on the Western Pacific Island of Yap, in the Federated States of Micronesia [3, 6, 7]. Two main strains of ZIKV were distinguished, the African and Asian lineages [8]. The symptoms of ZIKV infection include low fever, body aches, petechiae, myalgia, fatigue, joint pain, red eyes, headache and a maculopapular eruption, which resembles chikungunya and dengue diseases [9]. ZIKV infection of pregnant women can result in damage to the fetus, such as microcephaly and other brain malformations [10, 11]. In adults, severe neurological complications, such as Guillain-Barré syndrome may also occur [12].

The ZIKV is transmitted by mosquitoes of the genus *Aedes*, in Africa by the *A. africanus*, in Asia and the Americas by the *A. aegypti*. These species are epidemic or enzootic vectors [13–15]. The vertical transmission in humans occurs from an infected mother through the placenta to the fetus. In addition, ZIKV can be transmitted by blood transfusions and sexual intercourse [16, 17]. In 2016, the first human-to-human transmission was registered in Texas, USA. However, the most common route of ZIKV transmission is the bite of infected female mosquitoes (horizontal transmission) [18–22], which maintains the arbovirus cycles between *A. aegypti* and human populations.

The transovarial, or vertical transmission, is the virus transference from mother to a fraction of the offspring, and has been studied to understand virus persistence in nature and its epidemiological contribution in outbreaks [23–25]. Since the isolation of ZIKV from field-collected larvae in immature stage [25], and from male [26], which do not feed on blood (usually ingesting elaborate sap and nectar), there is evidence of ZIKV vertical transmission mechanism.

This work focuses on the progression of Zika virus infection in its vector *A. aegypti* through the analysis of the images obtained by transmission electron microscopy (TEM). We characterized the flow of ZIKV infection at the third day after infection. In addition, the ZIKV vertical transmission (from mosquitoes to their progeny) was also investigated by quantification of ZIKV RNA copies, using RT-qPCR technique in pools of orally infected females on 3 days after blood meal and in the pools of embryonated eggs laid by these females.

## Results

In order to demonstrate the route of ZIKV infection in *A. aegypti*, Liverpool strain starved females were orally infected by feeding, as described in Material and Methods. After 3 days, the experimental and control females had their midgut, fat body, ovary and head dissected. The tissues were fixed and processed for analysis by TEM as described in Material and Methods.

**Fig. 1:**
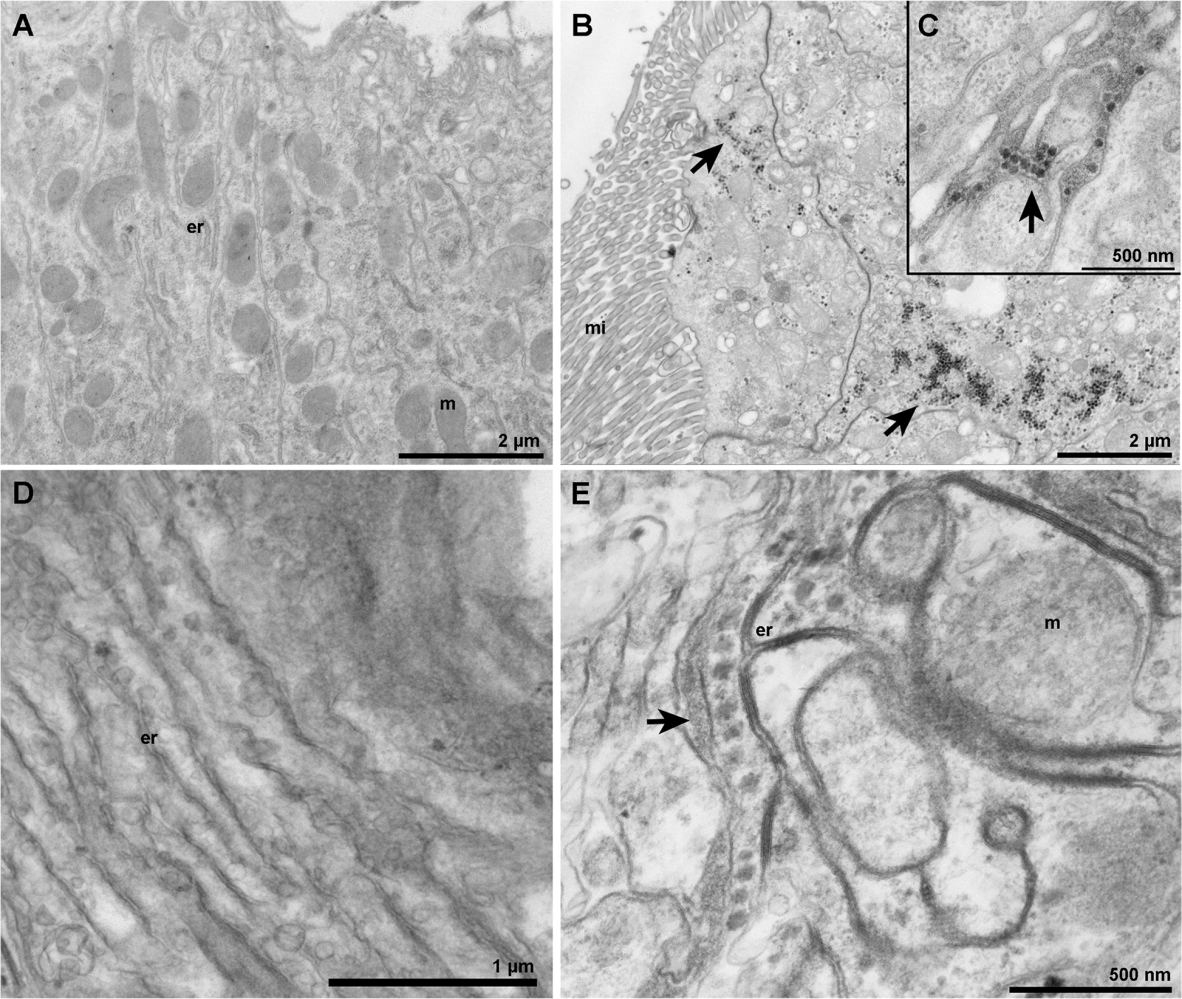
Transmission electron microscopy of mock and ZIKV infected midgut and greasy bodies from *A. aegypti*. **A** Panoramic image of polarized cells from the insect midgut showing unaltered organelles as mitochondria and ER; **B** Clusters of ZIKV (arrows) were observed in the tissue of infected mosquito; **C** Arrow points to a ZIKV-containing cisternae; **D** Fatty bodies tissue presenting ER profiles; **E** ER-containing ZIKV particles (arrow) in fatty bodies of infected mosquitoes. (ER) Endoplasmic reticulum; (m) mitochondria; (Mi) Luminal microvilli. Bars: **A-B** 2 μm; **C**,** E** 500 nm; **D** 1 μm

Mock and infected *A. aegypti* midguts are shown in Fig. 1 A and 1B, respectively. ZIKV particles migrating to the inner cells of the tissue are shown Fig. 1B. Cisternae containing ZIKV particles were also observed in this tissue (Fig. 1 C).

Mock-infected and ZIKV infected *A. aegypti* fat bodies are shown in Fig. 1D and E. In Fig. 1E, an ER-like structure harbors ZIKV immature particles. A reduced number of viral particles was observed in this tissue, when compared to the intestine (Fig. 1B-C).

**Fig. 2:**
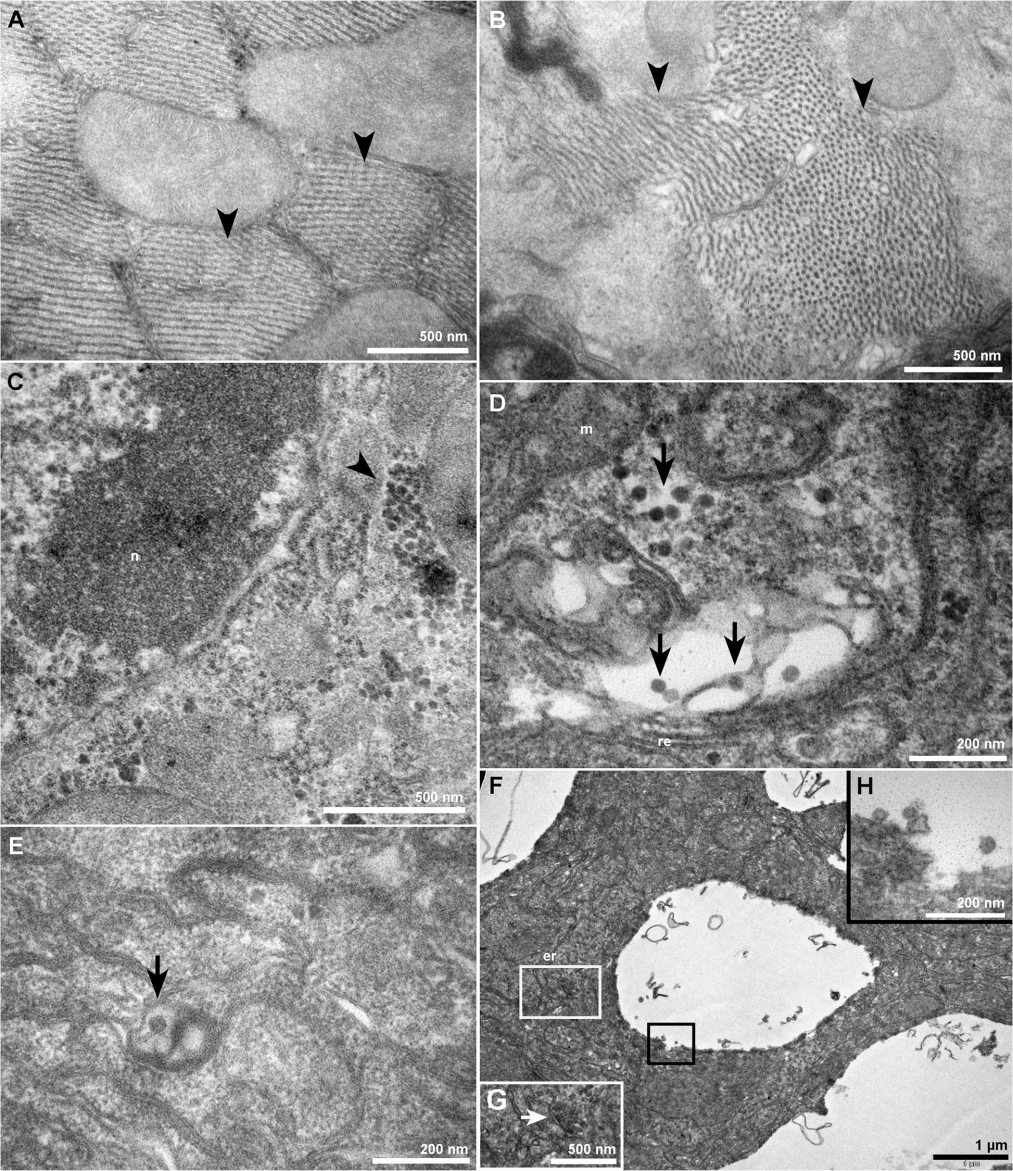
Transmission electron microscopy of mock and ZIKV infected head from *A. aegypti*. In non-infected insects, arrowheads point to muscular structures in the mosquito’s eyes longitudinally (**A**), and transversely (**B**) sectioned; (**C**) Granules of different sizes and amorphous shape (arrowheads) were also noted in the head of non-infected mosquitoes; Also in this tissue, viral nucleocapsids of 40 nm in diameter were observed surrounded by membrane profiles (arrows) (**D-E**), and within vacuoles (**F-H**). Bars: (**A-C**) 500 nm; (**D-E, H**) 200 nm; (**F**) 1 μm

In the head of non-infected mosquitoes, elongated muscular structures were found in the insect eyes zone (Fig. 2 A), acquiring a spherical shape when transversely sectioned (Fig. 2B). Clusters of dense granules from different sizes were also found in this tissue (Fig. 2 C). In the head of infected mosquitoes, however, ZIKV particles were clearly distinguished, surrounded by membrane profiles (Fig. 2D-E) or inside vacuoles (Fig. 2 F-H).

**Fig. 3:**
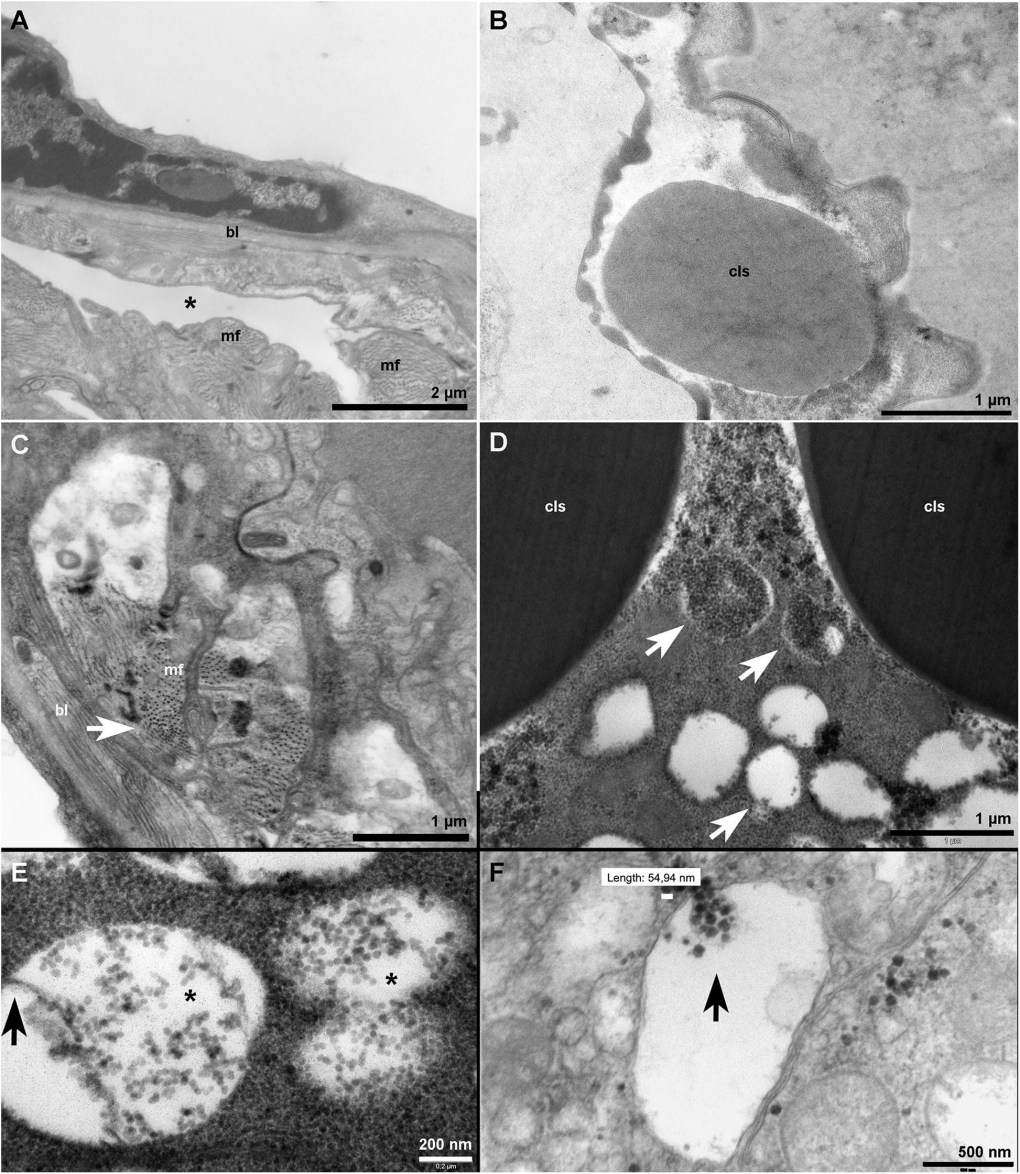
Transmission electron microscopy of mock and ZIKV infected ovaries from *A. aegypti*. In non-infected ovaries (**A**), asterisk marks the lumen of *A. aegypti* oviduct; (**B**) Chitin-like structure (cls) involved in *A. aegypti* egg formation was observed; (**C**) The same elongated electron-dense structures observed in the head tissue was present in the ovaries (arrow); Arrows in (**D**) point to vacuoles containing numerous virus particles, around the chitin-like structures; Most of these vacuoles displayed a detaching membrane of 20 nm in width (arrow) that seemed to contain the particles; **E-F** In other vacuoles, vesicles (arrow) seem to harbor the virus particles of 54 nm in diameter. Bars: (**A**) 2 μm; (**B-D**) 1 μm; (**E**) 200 nm; (**F**) 500 nm

The females of *A. aegypti* remained incubated for 3 days with free copula for the production of embryonated eggs. Mature ovaries of non-infected mosquitoes are shown in Fig. 3 A-B, where regions from the oviduct (Fig. 3 A) and dense structures resembling chitin involved in egg formation (Fig. 3B) were observed. Similar to what was observed in the head, transversely sectioned muscular zones showed circular shapes (Fig. 3 C). The infected tissue (Fig. 3D-F) displayed virus-like particles (VLPs) of approximately 50 nm in diameter around dense structures in the ovary of infected *A. aegypti*. A higher rate of VLPs was found in the ovary when compared to fat bodies and head tissues. Many VLPs-containing vacuoles were noted (Fig. 3E-F), and these particles seem to be enclosed by a membrane of 20 nm in thickness. The amount and localization of VLPs suggests that the infection of this tissue is subsequent to that of the midgut.

In order to confirm the presence of ZIKV in orally infected female mosquitoes, three pools of 3 individual mosquitoes in each, at the 3rd day post-infection, were subjected to individual RT-qPCR analysis, revealing a median of 5.5 × 10^3^ ZIKV RNA copies/female, while two pools of 100 laid eggs after embryogenesis period were estimated in 7.45 × 10^1^ (OIV3) and 7.14 × 10^1^ (OIV4) ZIKV RNA copies (Table 1).

**Table 1:** RT-qPCR of mosquitoes and eggs offspring

Description	Abbreviation	Mean Ct values	SD	N. of Copies/µL
Mosquitoes Liverpool (control) *pool-1* - *in vitro*	MO1	N/A	N/A	N/A
Mosquitoes Liverpool (control) *pool-2* - *in vitro*	MO2	N/A	N/A	N/A
Mosquitoes Liverpool (control) *pool-3* - *in vitro*	MO3	N/A	N/A	N/A
Mosquitoes Liverpool (ZIKV) *pool*-1 - *in vitro*	MO4	29,53	0,31	1,91E + 02
Mosquitoes Liverpool (ZIKV) *pool*-2 - *in vitro*	MO5	29,23	0,12	1,87E + 02
Mosquitoes Liverpool (ZIKV) *pool*-3 - *in vitro*	MO6	27,65	0,06	1,61E + 03
Eggs Liverpool control *pool-1* - *in vitro*	OIV1	N/A	N/A	N/A
Eggs Liverpool control *pool-2* - *in vitro*	OIV2	N/A	N/A	N/A
Eggs Liverpool (ZIKV) *pool-1* - *in vitro*	OIV3	34,80	0,19	7,45E + 01
Eggs Liverpool (ZIKV) *pool-2* - *in vitro*	OIV4	34,07	2,17	7,14E + 01
RNA Zika Virus	ZIKV	17,90	0,35	6,36E + 08
Plasmid ZIKV	PZ^− 4^	22,49	0,01	2,13E + 07
Plasmid ZIKV	PZ^− 2^	12,18	0,13	2,13E + 09
Nuclease free water (Blank of reaction)	H2O	N/A	N/A	N/A

## Discussion

Several studies showed the *Aedes* mosquitoes’ genus ability to transmit ZIKV in nature [15, 27–29]. Recent studies with chikungunya virus (CHIKV) showed virus spreading inside the mosquito intestine after the ingestion of CHIKV-containing blood [30]. Our study evaluates the progression of the ZIKV infection in *A. aegypti* after a 3-day period of ingestion of blood containing virus, as occurs naturally when mosquitoes feed, thus generating an infection similar to that in the wild. The ZIKV has some distinctive characteristics from other flaviviruses, such as the high virus titers when compared with other members of this genus. Besides, it presents fast dissemination and great medical importance, which makes the ZIKV an attractive model to verify the viral dissemination after the oral feeding with blood containing virus.

In the midgut tissue (Fig. 1B), viruses replicate rapidly and spread through the midgut cells. The viruses cross the midgut epithelium cells towards the inner layer of the tissue. We could observe the formation of ZIKV-filled cisternae (Fig. 1 C), demonstrating a well-established infection when compared to the control in Fig. 1 A. This rapid propagation has been reported for other arboviruses, such as CHIKV, that spread during the digestion of blood from the midgut epithelium to the basal lamina (BL) around the midgut [30].

The virus accumulation towards the BL has been previously reported to Western equine encephalitis virus (WEEV; Alphavirus) infection [31, 32]. More recently, Cui et al. (2019) reported the increase of ZIKV titers in the midgut of female mosquitoes 3 day after infected blood meal ingestion. The authors propose that midgut distention, due to meal ingestion, results in the enlargement of the BL, favoring viral egress from this infected tissue towards the secondary tissues [33].

In addition, Romoser et al. [34] suggested that arboviruses such as the Rift Valley fever virus (RVFV) penetrate at modified BL and infect the tracheo-muscular complex, which becomes a channel of dissemination from the midgut [32].

Fat body from both mock and infected mosquitoes showed large amounts of glycogen granules of varying sizes (Fig. 1D-E). This was also observed in mosquito tissue, by Martins et al. (2011) [35]. De novo synthesized virions were observed within vesicular structures within the ER of the infected tissue (Fig. 1E), while in the fat body of mock-infected mosquitoes only the occurrence of spreading profiles of ER was noted (Fig. 1D).

The region of the mosquito eyes compared in Fig. 2 A-B presents the accumulation of structures that, when transversely sectioned, assume a spherical shape. The proliferation of membrane profiles in infected mosquito tissue (Fig. 2 C-D) was expected, since the infection occurred for 3 days until the dissection of the mosquitoes. In general, the presence of flaviviruses in the salivary glands occurs around 5 days post-infection (dpi) [32, 34, 35]. Nevertheless, virus particles were scarce in the head organs of infected mosquitoes when compared to the ovaries (Fig. 3D-F), suggesting that ZIKV could not completely reach this tissue at 3 dpi.

The BL and other types of tissue were well preserved (data not shown). In the ovaries of infected females, virus spreading along the tissue may include the crossing of the ovary cells and the formation of ZIKV-containing vacuoles. Interestingly, the VLPs within these vacuoles seemed enclosed by additional membranes. It is important to note that, since the image was acquired from a tissue slice, it is possible that what was noticed as a vacuole, could be a membrane invagination.

Differently, vacuoles containing accumulated VLPs in the ovary suggest an advanced infection and an in-process dissemination, prior to other tissues due to the fact that this organ could exhibit the second larger amount of ZIKV particles, after the midgut.

The practice of hematophagy in *A. aegypti* females is essential for egg production [18] and allows the virus to spread by the bite of the infected insect. Blood proteins digested into amino acids and other compounds are taken up by the fat body, inducing vitellogenin (Vg) protein synthesis. Vg together with other yolk proteins are packaged into oocytes [19, 20]. We hypothesize that this process may contribute to the abundance of ZIKV-containing vacuoles in the ovarian tissue (Fig. 3D-F), which would enable the vertical transmission of virus.

The oocyte, then, passes through the spermatheca where they are fertilized. The female lays eggs between the 3rd and 4th days after blood meal. In the same period, the pathogens that infected the mosquitoes during blood meal, move across the midgut, spread through the hemocoel, invading organs such as the fat body and ovaries as described in this study. This increases the temporal chance to the vertical transmission.

After egg laying, the egg outer membrane, composed mainly by proteins and chitin material, becomes rigid throughout sclerotization. This process provides mechanical protection from the action of pathogens, insecticides, and prevents the excessive loss of water by the embryo contained inside [21, 22]. However, this could probably also preserve, in the case of ZIKV infection, the viruses inside the eggs.

Once the ovaries are infected prior to other organs, chances of vertical transmission increase - in an analogue way to what occurs in humans when mothers transmit the virus to the embryos during the pregnancy [36–39]. This type of transmission was also observed for other arboviruses, including DENV, CHIKV, WNV and YFV [40–51].

The results presented here can contribute to the understanding of the ZIKV propagation throughout the *A. aegypti* tissues. In addition, our data suggest that the female mosquito is able to transmit the ZIKV to its offspring, due to the large amount of VLPs found in the ovaries. RT-qPCR data from female mosquitoes and eggs corroborated our hypothesis (Table 1), since the ZIKV genome was detected in eggs laid from infected females. The infection of *A. aegypti* mosquitoes by feeding in laboratory, performed by other research groups, also indicates the existence of vertical transmission of ZIKV through the detection of the virus in larvae, pulp and in mosquitos’ offspring of infected females [52, 53]. While previous studies detected ZIKV in mosquitoes and larvae collected in the wild [23–26], our work showed the detection of ZIKV in eggs of *A. aegypti*. If throughout the oogenesis period, the ovaries contain large quantities of viral particles, females would lay the already infected eggs. Thus, transovarial or vertical propagation, can be an important route of ZIKV transmission in addition to infection by a blood meal. The laid egg could be a virus reservoir, since its durability in nature is greater than the mosquito longevity [54].

## Conclusions

In conclusion, our study approached the electron microscopy of ZIKV in *A. aegypti* tissues 3 days after virus ingestion through oral feeding. Together with data obtained by RT-qPCR analysis, our work corroborates the spreading of ZIKV through the mosquito tissues that occurs from the intestine towards other organs of the organism. The high degree of infection in the ovary, reinforced by VLPs accumulation inside vacuoles when compared to the other tissues analyzed, suggest that the ovary is probably the second organ to be infected in this course. Nevertheless, future studies are needed to determine this sequence of infection, and the possibility that mosquitoes’ eggs are already carrying ZIKV in the early stages of the mosquito infection, even before the insect is able to transmit the virus to new hosts during a blood meal.

## Methods

### Cells and virus

Vero cells (African green monkey kidney, ATCC CCL-81) were cultured at 37 °C with 5 % CO_2_ in Dulbecco’s modified Eagle’s medium (DMEM) (Life Technologies, USA) supplemented with 5 % fetal bovine serum (Life Technologies, USA), 50 IU/mL penicillin and 50 µg/mL streptomycin (Life Technologies, USA).

ZIKV (MR 766 strain) was propagated in Vero cells, and viral stocks supplemented with 10 % glycerol were kept at -70 °C. The virus titer was determined by plaque assay, and stock viruses were first propagated in Vero cells in 25 cm² bottles for 5 days, when the viral suspension was removed and added to Vero cells in 175 cm² bottles. The viral suspension was clarified by centrifugation (5000 x g for 5 min at 4 °C) and concentrated by vivaspin (GE Healthcare, USA) following manufacturer’s procedures adapted protocol [55]. The particles were further purified in a gradient of 10 to 35 % potassium tartrate (126.308 G for 16 h at 4 °C in a Beckman SW55 Ti rotor), following an adapted protocol [56].

### Mosquito rearing

*A. aegypti* (Liverpool strain) were maintained in a mosquito rearing facility at the Federal University of Rio de Janeiro (UFRJ), Brazil, under a 12 h light/dark (LD) cycle at 28 °C and 70∼80 % relative humidity. Larvae were raised in plastic trays containing 1 L of previously filtered water, and fed with 0,1 g of powdered dog chow. Tray water was changed every two days. After pupation, mosquitoes were transferred to plastic cages, allowed to emerge, and adults were fed with a sucrose 10 % solution *ad libitum*.

### Mosquito infection and dissection

Plastic cages containing 200 *A*. *aegypti* mosquitoes (100 males and 100 females – five days old) were artificially fed with a 1:1 mix of heparinized rabbit red blood cells and DMEM culture medium containing ZIKV (infected blood meal) at a final concentration of 2,5 × 10^9^ PFU/mL. Control mosquitoes (Mock) were fed with a blood meal without virus. Feeding was performed using water-jacketed artificial feeders maintained at 37 °C and sealed with parafilm membranes for approximately 1 h, inside a Biosafety level-2 (BSL-2) insectary facility. The insects were starved for 12 h prior to feeding. After feeding, poorly or unfed mosquitoes were removed from the cages in all the experiments. Mosquitoes were then maintained under BSL-2 insectary conditions (28 °C, LD 12:12, 70 % humidity) with sucrose 10 % solution *ad libitum*. Pools of 10 female midguts, ovaries, head and fat body were dissected 3 days post-blood meal, transferred to a glutaraldehyde 2 mM solution in sodium cacodylate 0,1 mM buffer, pH 7.2. A wet paper cup was used for the females to lay their eggs, and the eggs from the first oviposition cycle were collected for the analysis of ZIKV vertical transmission.

### Transmission Electron Microscopy

Three days after feeding with ZIKV, mosquitoes tissues such as midgut, fat body, ovary and head were fixed for 2 h at room temperature, in 2.5 % glutaraldehyde in 0.1 M cacodylate buffer (pH 7.2), and post-fixed at room temperature in 1 % OsO_4_ / 0.8 % potassium ferrocyanide for 1 h. Samples were gradually dehydrated in ethanol and flat-embedded in Polybed resin (Polysciences®). Ultrathin Sect. (60 nm thick) were stained with uranyl acetate and lead citrate [57]. The ultra-thin sections were observed using a transmission electron microscope (TEM Jeol 1200 and Morgani FEI company).

### ZIKV Detection and Quantification by RT-qPCR

Entire female mosquitoes and eggs used as experimental samples were processed for RNA extraction with Trizol (Invitrogen, Carlsbad, California, USA) and mechanical maceration, as instructed by the manufacturer. Reverse transcription was performed together with quantitative one-step PCR (GoTaq® RT-qPCR Systems, Promega, Madison, Wisconsin, USA), following the manufacturer’s instructions. qPCR assay for ZIKV was performed with primers set (5’-GTGTAAACCCTTGGGAGGTTT-3’ forward and 5’-AAGTTGGTAGCAAAGGAGATGGC-3’ reverse). Standard curve used was from CprM-ZIKV Gene Fragments dilutions, ranging 300,000 to 3 copies. All tests were performed in triplicate. BIO-RAD CFX96 touch instrument was used (BIO-RAD, Hercules, Califórnia, EUA), following the manufacturer’s instructions.

## Data Availability

Data is available on request to the authors.

## Abbreviations

BL: basal lamina
BSL-2: Biosafety level – 2
CHIKV: chikungunya virus
DENV: dengue virus
DMEM: Dulbecco's modified Eagle's medium
ER: Endoplasmic Reticulum
LD: light/dark
PAHO: *Pan-American Health Organization*
RVFV: Rift Valley fever virus
TEM: Transmission Electron Microscopy
USA: United States America
WEEV: Western equine encephalitis virus
WHO: World Health Organization
WNV: West Nile virus
YFV: yellow fever virus
ZIKV: Zika virus
